# Islet Amyloid Polypeptide: Structure, Function, and Pathophysiology

**DOI:** 10.1155/2016/2798269

**Published:** 2015-11-15

**Authors:** Rehana Akter, Ping Cao, Harris Noor, Zachary Ridgway, Ling-Hsien Tu, Hui Wang, Amy G. Wong, Xiaoxue Zhang, Andisheh Abedini, Ann Marie Schmidt, Daniel P. Raleigh

**Affiliations:** ^1^Department of Chemistry, Stony Brook University, Stony Brook, NY 11794-3400, USA; ^2^Diabetes Research Program, NYU School of Medicine, 550 First Avenue, New York, NY 10016, USA; ^3^Research Department of Structural and Molecule Biology, University College London, Gower Street, London WC1E 6BT, UK

## Abstract

The hormone islet amyloid polypeptide (IAPP, or amylin) plays a role in glucose homeostasis but aggregates to form islet amyloid in type-2 diabetes. Islet amyloid formation contributes to *β*-cell dysfunction and death in the disease and to the failure of islet transplants. Recent work suggests a role for IAPP aggregation in cardiovascular complications of type-2 diabetes and hints at a possible role in type-1 diabetes. The mechanisms of IAPP amyloid formation *in vivo* or *in vitro* are not understood and the mechanisms of IAPP induced *β*-cell death are not fully defined. Activation of the inflammasome, defects in autophagy, ER stress, generation of reactive oxygen species, membrane disruption, and receptor mediated mechanisms have all been proposed to play a role. Open questions in the field include the relative importance of the various mechanisms of *β*-cell death, the relevance of reductionist biophysical studies to the situation *in vivo*, the molecular mechanism of amyloid formation *in vitro* and *in vivo*, the factors which trigger amyloid formation in type-2 diabetes, the potential role of IAPP in type-1 diabetes, the development of clinically relevant inhibitors of islet amyloidosis toxicity, and the design of soluble, bioactive variants of IAPP for use as adjuncts to insulin therapy.

## 1. Introduction

**Figure 1:**
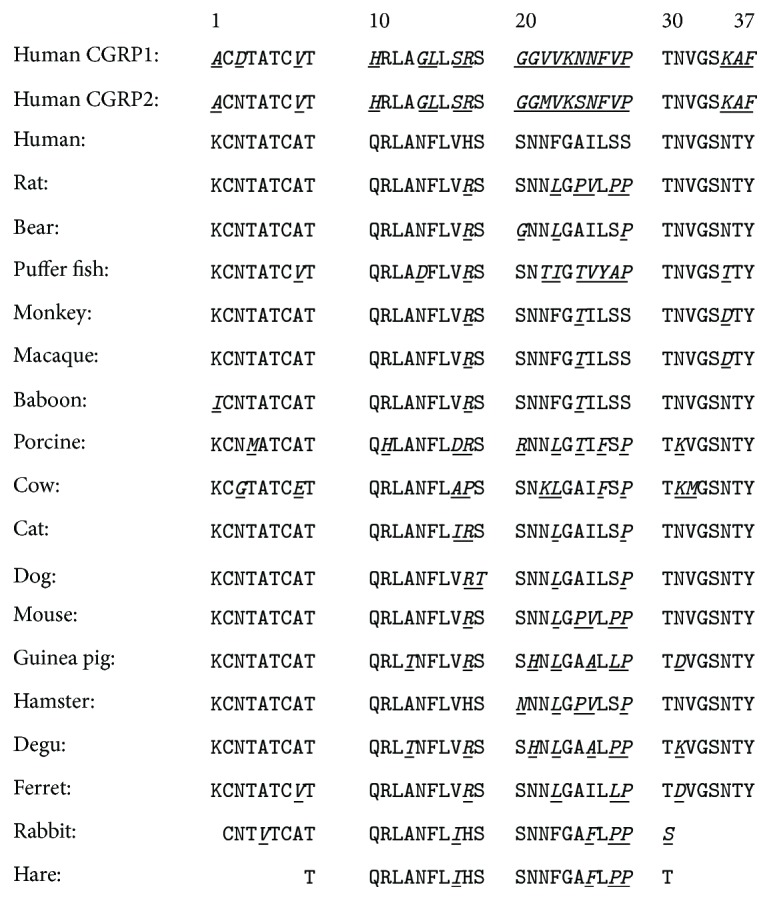
Primary sequences of human CGRP and of IAPP from different species. Residues that differ from the human IAPP sequence are in italics and underlined. The biologically active sequences all contain a disulfide bridge between Cys-2 and Cys-7 and have an amidated C-terminus. Primates and cats have been reported to form islet amyloid while, rodents, and dogs do not. Ferret and porcine IAPP are reported to be significantly less amyloidogenic than human IAPP. The ability of cow, bear, and puffer fish IAPP to form amyloid have not been investigated. Islet amyloid is found in the degu, a rodent, but it is derived from insulin, not from IAPP. Only partial sequences are available for rabbit and hare.

Hyaline lesions in the pancreas were first described more than 110 years ago by Opie [1] and were later identified as amyloid. The deposits were originally assumed to be composed of insulin, fragments of insulin, or proinsulin, but 85 years after Opie's initial observation two groups independently identified the major protein component of islet amyloid as a 37-residue polypeptide neuropancreatic hormone, now known as islet amyloid polypeptide (IAPP) or amylin [2, 3] (Figure 1). IAPP was subsequently shown to play an adaptive role in metabolism and glucose homeostasis, helping to control gastric emptying, glucose homeostasis, and suppression of glucagon release and helping to regulate satiety [4–6].

**Figure 2:**
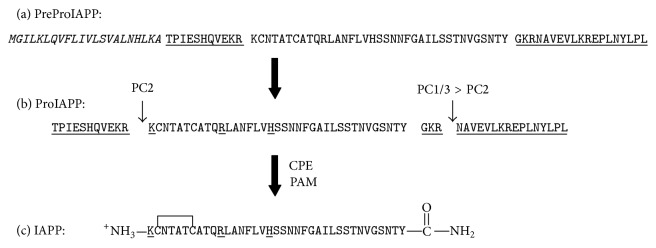
Processing of human PreProIAPP to produce mature IAPP. (a) The primary sequence of the 89-residue human PreProIAPP. The 22-residue signal sequence is shown in italics; the N- and C-terminal proIAPP flanking regions are underlined. (b) Primary sequence of the 67-residue proform of human IAPP. Pro-hIAPP is cleaved by the prohormone convertases PC(1/3) and PC2 at two conserved dibasic sites, indicated by arrows. The amidated C-terminus is produced after further processing by CPE/PAM. (c) The sequence of the mature 37-residue human IAPP. The biologically active peptide has an amidated C-terminus and a disulfide bridge between Cys-2 and Cys-7.

IAPP has been found in all mammals studied to date and the sequence is strongly conserved although there are interspecies variations and these correlate with the ability to form amyloid* in vivo* (Figure 1). The hormone is synthesized as an 89-residue preprohormone and, after cleavage of the signal sequence, the 67-residue proform is processed in the Golgi and in the insulin *β*-cell secretory granule to yield the mature hormone (Figure 2) [7, 8]. IAPP is stored with insulin in the granule and is released in response to the stimuli that lead to insulin secretion [9–11].

In this review we discuss the physical chemical properties of IAPP, its normal function, the structure of the monomer, and the amyloid fibril and then focus on amyloid formation and the pathophysiology of IAPP. We also touch upon efforts to design analogs of human IAPP (hIAPP) suitable for use as adjuncts in insulin therapy. It is not possible to cover all topics and all of the recent developments in IAPP research in a limited review and the reader is referred to other articles in this issue for coverage of other topics and for alternative views. We do not discuss efforts at inhibitor design as they are described in other articles in this issue and have been reviewed elsewhere [12]. A number of review articles have been published in recent years which provide additional information on various aspects of the biology and biophysics of IAPP [4–6, 12–15].

Much has been learned about the role of IAPP in glucose metabolism, and the role of islet amyloidosis in metabolic disease, but there is still much that is unclear. The site of initiation of amyloid formation* in vivo* is controversial. The mechanism(s) of IAPP amyloid formation* in vivo* and* in vitro* are still not understood nor are the factors which trigger islet amyloidosis in type-2 diabetes (T2D). The nature of the toxic species generated during IAPP amyloid formation is not well defined and the mechanisms of *β*-cell death are not completely understood. The possible role of IAPP aggregation in the complications of diabetes has yet to be fully defined and the potential role of IAPP in type-1 diabetes remains to be elucidated [16–18]. Unfortunately, inhibitors of IAPP *β*-cell toxicity are less well developed than for other amyloidogenic proteins and no clinically relevant inhibitors of islet amyloidosis toxicity have yet been described. There is also interest in developing bioactive, nontoxic analogs of IAPP with improved solubility for use as adjuncts to insulin therapy and for potential coformulation with leptin.

## 2. The Physical Chemical Properties of IAPP and the Importance of the 20–29 Region in Amyloid Formation

hIAPP is a relatively hydrophobic polypeptide but contains several positively charged residues, Lys-1, Arg-11, and, depending upon the pH, His-18 (Figure 1). There are no acidic residues in the molecule and the C-terminus is amidated; thus its pI is above the pKa of the Tyr and Lys residues. The polypeptide is positively charged at and below physiological pH with a net charge ranging from 2 to 4 depending upon the pKa's of the N-terminus and His-18 and the pH. The net positive charge on the molecule is important for interactions with negatively charged, nonphysiological model membranes and for interactions with sulfated proteoglycans of the extracellular matrix. The sequence of hIAPP contains an unusually large number of Asn and Ser/Thr residues for its size, 6 and 10, respectively. There are three aromatic residues including a conserved C-terminal Tyr, a conserved Phe at postion-15, and a second Phe at postion-23.

IAPP belongs to the calcitonin related peptide family which is comprised of adrenomedullin, *α*- and *β*-calcitonin gene-related peptide (CGRP), intermedin, and calcitonin. The peptides all share key posttranslational modifications; they all have an amidated C-terminus and contain an intramolecular disulfide bridge near the N-terminus (Figure 1). IAPP is most similar to CGRP. The two peptides have reasonable sequence similarity but diverge the most within the segment corresponding to residues 20 and 29 [19]. hIAPP is aggressively amyloidogenic* in vitro*, but CGRP does not form amyloid. These observations led to the initial hypothesis that the sequence of the 20 to 29 region dictates the ability of IAPP to form amyloid and were supported by studies with ten residue peptides derived from residues 20 to 29 of hIAPP [19, 20]. Not all mammals form islet amyloid; notably mice and rats do not [19, 21]. Comparison of the rat/mouse sequence to the sequence of hIAPP, together with early* in vitro* experiments, appeared to confirm the hypothesis that the ability to form amyloid is controlled by the identity of the 20–29 segment [20, 21]. hIAPP and rat IAPP (rIAPP) differ at six positions and five of these are between residues 23–29. Of particular note, the rat sequence contains three Pro residues at positions 25, 28, and 29, while the human sequence has none (Figure 1). Pro is highly energetically unfavorable in a *β*-sheet and is a well-known disrupter of secondary structure. Consequently, the inability of rat IAPP to form amyloid has been attributed to the Pro substitutions [21]. These important early studies led to the view that the ability of IAPP to form amyloid* in vitro* and* in vivo* is determined by the primary sequence in the 20–29 region; however the situation is more complex.

Other fragments, in addition to the 20–29 segment of hIAPP, were subsequently shown to be capable of forming amyloid in isolation, arguing that the 20–29 region is not the only amyloidogenic segment of the polypeptide. These include peptides comprised of residues 30–37, 8–20, and 10–19 and even smaller fragments from the 10–19 region [22–25]. The work with the smaller fragment led to the suggestion that this region of the chain is likely important for formation of initial hIAPP hIAPP contacts during aggregation [25]. Peptide array studies, in which a family of overlapping peptides that span the entire region of hIAPP were tested for hIAPP binding, confirmed the importance of this region. Subsequent X-ray crystallographic structural studies with a truncated hIAPP maltose binding protein fusion construct revealed pairs of hIAPP molecules making interprotein contacts in this region [26]. Interestingly, the region of hIAPP that appears to be important for self-contacts also appears to be important for interactions with insulin and with the A*β* peptide of Alzheimer's disease [27, 28].

Studies on intact hIAPP also indicate that the 20–29 segment is not the sole amyloidogenic determinant. Multiple Pro substitutions outside of the 20–29 region abolish amyloid formation by hIAPP and replacement of Asn-14 or Asn-21 has been reported to do so as well [29, 30]. Conversely, substitution of residues 18, 23, and 26 in rIAPP by the corresponding amino acids of hIAPP led to a weakly amyloidogenic polypeptide even though it still contained the 3 Pro residues of rIAPP [31]. Thus, the 20–29 sequence cannot be the only factor governing amyloid formation, but there is no doubt that it is important and single proline substitutions within the 20–29 segment have been shown to significantly reduce the amyloidogenicity of hIAPP as have double N-methyl modifications in this region [32–34].

## 3. The Structure of the IAPP Amyloid Fibril

High resolution models of the IAPP amyloid fibril have been developed based upon solid state NMR studies, and on X-ray diffraction studies of microcrystals of small peptide fragments of hIAPP which form steric zippers. Although they differ in their details, mainly in the location of the C-terminal *β*-strand, they are broadly similar; each is made up of two symmetrically related columns of IAPP monomers (Figure 2) [35, 36]. Monomers within each column pack on top of each other to generate a U-shaped structure with the *β*-sheet hydrogen bonds between adjacent IAPP molecules in one column. Each column contains two in-register parallel *β*-sheets. In the solid state NMR model, the N-terminal strand encompasses residues 8 to 17 and the C-terminal strand residues 28 to 37. The fragment based model differs from the NMR model in the location of the C-terminal *β*-sheet; it places residues 23 to 37 in the C-terminal *β*-sheet and residues 8 to 17 in the N-terminal *β*-sheet. Two structures were proposed based on the solid state NMR data, both of which are consistent with the experimental restraints. The major differences between the two are the register of the side chains. In one structure, Arg-11, Ala-13, and Phe-15 are all solvent-exposed, and in the other they project into the core of the fibril. Burial of a charged Arg side chain will be energetically unfavorable and the structure in which it is exposed seems more likely.

Independent amide H/D exchange measurements and two-dimensional infrared (2DIR) studies are largely consistent with the NMR model. Amide H/D exchange rates are sensitive to H-bonding and have recently been used to study amide proton solvent protection in hIAPP amyloid fibrils. The data are consistent with an N-terminal *β*-strand made up of residues 8 through 18 and a C-terminal strand comprised of residues 26 to 37 [37]. 2DIR line widths are sensitive to local dynamics and can be combined with molecular dynamics simulations to probe protein structure. A combined experimental 2DIR and computational study of hIAPP fibrils has been reported and the pattern of experimental line widths is consistent with predictions based upon the solid state NMR model [38].

Does the fact that the solid state NMR and fragment based models differ from one another mean that one is correct and one must be wrong? Not necessarily. It is important to emphasize that both structures are models based on, and consistent with, separate sets of experimental data which are sufficient to constrain the models but not to completely define a precise, three-dimensional, high resolution structure. Given the very different data used to construct the models, it is striking and reassuring that they share many common features. In addition, it is important to bear in mind that amyloid fibrils are polymorphic and thus the alternative structures could well represent different polymorphs [39–42].

An interesting alternative model, which differs from both the NMR and the fragment based model, has been proposed based upon EPR studies conducted with a set spin labeled variants of hIAPP incorporated into the polypeptide via Cys mutations. The method is more perturbing than the NMR or crystallographic approach since the spin labels could represent a large perturbation at a particular site, given the necessity of introducing a Cys and given the size of the spin label and its linker. However, the study involved an impressive number of variants and included analysis of electron microscopy data as well [43]. The model shares several of the general features of the NMR and fragment based models in that each IAPP molecule bends into an approximate U-shaped structure and contains two *β*-strands; in the case of the EPR model, these are made up of residues 12 to 19 and of 31 to 36 with residues 7 to 10 in a transition region. The location of ordered secondary structure is broadly consistent with that proposed from the NMR studies. The key difference between the EPR based model and the others is that the two strands in an IAPP monomer are staggered by about 15 Å with respect to each other in the EPR model; the staggered relationship leads to a left-handed twist. The authors proposed that the EPR structure could represent an alternative polymorph.

**Figure 3:**
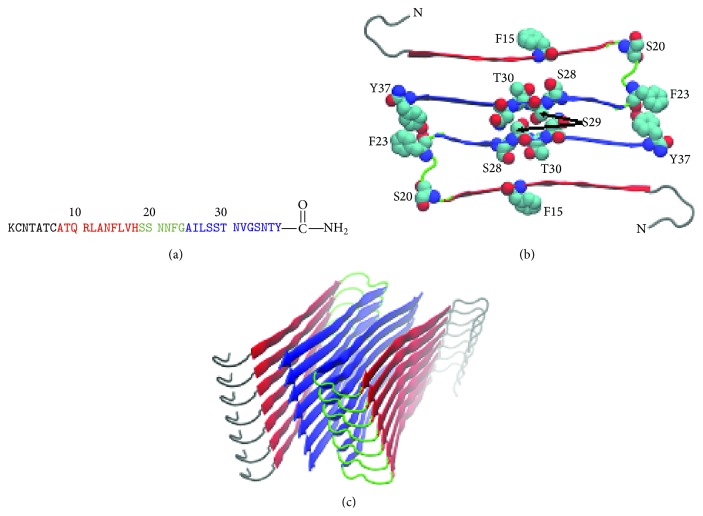
A model of the hIAPP fibril based on crystal structures of small peptide fragments of hIAPP. (a) Primary sequence of human IAPP. Residues color-coded red are found in the first *β*-strand in the fibril; those colored blue in the second *β*-strand and the ones located in the partially ordered loop that connects the two stands are colored green. The first seven residues are believed to be outside of the structured core of the fibril and are color-coded black. (b) Top down view of the model with several key residues shown. The N-termini are labeled. (c) View rotated by 90° showing the arrangement of the two stacks as a ribbon diagram.

Strikingly, most of the 20–29 segment is not part of the ordered *β*-sheet structure in the NMR and EPR based models but rather forms a loop which links the two *β*-strands (Figure 3). A loop should be able to accommodate mutations, making it unclear why mutations in this region have such a dramatic impact on amyloid formation. Time resolved 2DIR studies provide a possible resolution of the apparent conundrum [44]. Under the conditions of the 2DIR studies, a transient “nonnative” intermediate is formed that has parallel *β*-sheet structure localized in residues 23–27. This structure must ultimately be disrupted to form the loop which is found in the fibril. The location of the transient *β*-sheet offers an explanation for the sensitivity of IAPP amyloid formation to some of the substitutions within the 20–29 region [44]. Along these lines, stabilization of turn structures in the A*β* peptide of Alzheimer's disease can enhance significantly the rate of amyloid formation [45]. The structure derived from the fragment model can rationalize the sensitivity of amyloid formation to substitutions within the region of residues 24 to 29. This segment is well ordered in the model and both Ser-28 and Ser-29 are involved in critical contacts (Figure 3), rationalizing why the three Pro residues in rat IAPP impact amyloid formation.

## 4. Spontaneous Deamidation of Asn Residues Can Impact the Ability of hIAPP to Form Amyloid

The six Asn residues in hIAPP render the molecule susceptible to deamidation. Spontaneous Asn deamidation is one of the most common nonenzymatic posttranslation modifications of proteins and is believed to play a role in amyloid formation by other polypeptides [46]. Deamidation of Asn occurs via formation of a cyclic succinimide intermediate which leads to the conversion of an Asn residue into L or D Asp or L or D iso-Asp. The final product depends upon how the ring is opened. In all cases a neutral residue is replaced by a negatively charged residue which reduces the net charge of hIAPP and could thus reduce its solubility. Generation of iso-Asp introduces another rotatable bond in the peptide backbone which will impact its conformational propensities, while generation of a D amino acid alters significantly the allowed regions of the *ϕ*-*φ* plot. Asn deamidation has been shown to accelerate hIAPP amyloid formation* in vitro* and to lead to changes in the morphology of hIAPP amyloid fibrils [47]. Deamidation has also been shown to promote amyloid formation by otherwise nonamyloidogenic peptide fragments of hIAPP [48]. Of practical concern, deamidation is sensitive to the choice of buffer and this should be kept in mind when conducting experiments that involve incubating the polypeptide for significant lengths of time.

It is not known if deamidation plays a role in islet amyloid formation* in vivo*. A significant challenge with any potential study will be to define a causal relationship. The observation of deamidated material in isolated islet amyloid deposits would not prove that deamidation leads to amyloid formation since deamidation could have occurred after formation of the amyloid fibril. An issue for any biophysical study is the challenge of characterizing the highly heterogeneous ensemble that can arise from six potential sites for deamidation with five potential substitutions at each site (the normal Asn residue plus the 4 possible deamidation products).

## 5. Mutational Analysis of Amyloid Formation by IAPP

The only natural mutation found in the mature sequence of hIAPP is a Ser to Gly missense mutation at position 20. This mutation, which is found at low levels in certain Asian populations, has been proposed to lead to a slightly higher risk of diabetes, although the statistical significance has been questioned [4, 49–52]. The mutation accelerates amyloid formation* in vitro*, but the mechanism by which it does so is unknown. Stabilization of globular proteins or acceleration of their folding rate by substitution of an L-amino acid with Gly is often due to the fact that Gly can relieve steric clashes and/or adopt “left handed” conformations with a positive *ϕ*-backbone dihedral angle that are energetically unfavorable for an L-amino acid. However, the side chain of Ser-20 makes no obvious clashes in the existing models of IAPP amyloid fibrils and it adopts a normal *ϕ*-backbone dihedral angle. In addition, Ser-20 is located in the loop/bend region between the two *β*-strands in all of the models of hIAPP amyloid fibrils. The reason for the significant enhancement in the rate of amyloid formation by the Ser-20 to Gly mutation of hIAPP is still unknown.

Quantitative mutational studies of amyloid fibril stability and of the kinetics of amyloid formation are much more challenging than studies with soluble, monomeric, globular proteins. There are rigorous, well-established methods for determining the stability of soluble proteins, but this is not always the case for amyloids. Solubility measurements give simple interpretable apparent free energies, if the process is reversible, if the soluble phase is composed of monomers, and if activity effects can be ignored, but it is difficult to verify these assumptions. An added complication is that mutations can lead to different polymorphs and might alter the mechanism of self-assembly. Furthermore, studies that report that a mutation abolishes amyloid formation may have simply not examined the protein for a long enough time. In spite of these caveats, mutational analysis of amyloid formation has provided useful insight and systematic studies, such as proline and alanine scans, have been reported for a number of amyloidogenic proteins, but not for hIAPP.

No systematic analysis of all of the positions of IAPP has been reported, although a number of mutational studies have been conducted [12, 30, 51–55]. It can be difficult to compare different studies since a range of conditions have been used and the rate of IAPP amyloid formation is sensitive to small changes in buffer composition, temperature, added salt, pH, the degree of agitation, and even the volume of sample used in experiments. Residual TFA from HPLC purification can affect amyloid formation and issues with lot to lot variability of ostensibly pure commercial IAPP have been reported [56]. A further complication arises from the fact that many studies have made use of a truncated fragment of IAPP which lacks the first seven residues (IAPP_8–37_). These residues are outside of the ordered amyloid core in both the NMR and X-ray models, but they might still affect the stability of the amyloid fibers. If nothing else, the truncation removes the charge on the side chain of Lys-1 and, depending upon whether or not the N-terminus is acetylated, the charge on the N-terminus as well. These considerations mean that considerable caution needs to be employed when comparing data generated in different laboratories. Unfortunately some papers do not provide all of the details required to repeat measurements.

hIAPP contains three aromatic residues: Phe-15, Phe-23, and Tyr-37. Phe-15 and Tyr-37 are strictly conserved amongst known IAPP sequences while Phe-23 is often replaced with Leu (Figure 1). Aromatic-aromatic, hydrophobic-aromatic, and aromatic-cation interactions have been proposed to be important in amyloid formation. Early studies, involving Ala scanning of short peptides derived from IAPP, supported this conjecture [57, 58]. Subsequent studies that employed more conservative aromatic to Leu substitutions revealed that aromatic residues were not required for amyloid formation by the full length polypeptide, although mutation of the aromatic residues impacts the rate of self-assembly [54, 55, 59, 60]. For example, replacement of all three of the aromatic residues in hIAPP by Leu leads to 5- to 8-fold slower amyloid formation [54].

A systematic examination of the role of different Asn residues in hIAPP in amyloid formation and assembly has also been reported [30]. The authors used the approximately isosteric substitutions of Leu for Asn and found that substitution of different Asn residues had drastically different consequences on amyloid kinetics. The truncated 8–37 hIAPP fragment was used as background in this study. The Asn14Leu and Asn21Leu mutants did not form amyloid on the experimental timescale of these studies.

Asn to Leu mutants offer a nice example of the value of using isosteric substitutions. The Leu side chain has approximately the same size and shape as Asn, but cannot hydrogen bond and is nonpolar. This allows a simpler interpretation of the data than would experiments involving less conservative replacements. A similar approach could be used to probe the role of the different Thr residues via Val substitutions. Isosteric replacement of other residues in hIAPP requires nongenetically coded amino acids. The polypeptide can be prepared by solid phase peptide synthesis making such studies possible. For example, substitution of Ser with 2-aminobuytric acid represents an isosteric replacement and would allow the role of the OH group to be probed. This is of interest because Ser-28 and Ser-29 are located at the interface of the two symmetrically related columns of hIAPP monomers in the fibril structure and are involved in networks of hydrogen bonded interactions (Figure 3). In addition, Ser-19 and Ser-20 are highly conserved in known IAPP sequences and Ser-34 are strictly conserved, making them interesting targets for future analysis (Figure 1).

The literature on IAPP mutations has been critically reviewed in 2013 and, in the interest of space, we refer the interested reader to that work for a more detailed discussion [12]. However, some additional mutants have been analyzed since then and we briefly summarize the new results. The role of the amidated C-terminus has been examined, as has the role of His-18 [60]. NMR studies of a nonphysiological variant of hIAPP with a free C-terminal carboxyl group provided evidence for intermolecular interactions involving His-18 and Tyr-37 at pH 5.5 and it was suggested that these interactions play a role in the early stages of amyloid formation by hIAPP. However, a subsequent study revealed that mutants which were designed to disrupt the putative His-Tyr interaction actually accelerated amyloid formation, indicating that the interaction is not essential for amyloid formation [60]. Replacement of His-18 with either a Gln or Leu significantly accelerated amyloid formation. Analysis of the His-18 Gln mutant revealed that the rate of IAPP amyloid formation was still pH dependent between pH 5 and 8, thereby showing that the charge state of the N-terminus is an important factor modulating the rate of amyloid formation, even though the N-terminal region of IAPP is not part of the core *β*-sheet structure. Amdiation of the C-terminus was shown to accelerate IAPP amyloid formation relative to the variant with a free C-terminus, even though amidation increased the net charge on the polypeptide.

## 6. IAPP Is Synthesized as a Preprohormone

IAPP is synthesized as a 89-residue preproform (Figure 3) [7]. The first 22 amino acids constitute the signal sequence and the next 67 amino acids are the proform (proIAPP). The N- and C-terminal flanking regions of proIAPP are cleaved by the prohormone convertases PC2 and PC1/3 [7]. ProIAPP is processed in the Golgi and in the insulin secretory granule [7, 8]. Amidation of the C-terminus is a multistep process. The first C-terminal cleavage leaves a Gly-Lys-Arg tripeptide sequence as the new C-terminus. The dibasic Lys-Arg pair at the C-terminus is removed by carboxypeptidase and the Gly acts as the nitrogen donor for amidation of the C-terminal Tyr by the peptidyl amidating monooxygenase complex (PAM). Disulfide bond formation leads to mature IAPP (Figure 3). Incorrect processing of proIAPP has been proposed to play a role in islet amyloid formation* in vivo*, but relatively little work has been done* in vitro* on amyloid formation by partially processed IAPP [8, 61–65].

Mature IAPP is stored in the insulin secretory granule and is found in the halo region of the granule, while insulin is located in the dense core. The concentration of IAPP in the granule is noticeably lower than that of insulin, about 1%-2% of the insulin level, but it is still much higher than that required to promote rapid amyloid formation* in vitro* [66, 67]. Thus, there must be factors that prevent the irreversible aggregation of IAPP in the granule. The low pH environment of the granule contributes since the rate of IAPP amyloid formation is slower at intragranule pH [60, 68–70]. Soluble insulin is one of the most potent inhibitors of IAPP aggregation and may play a role in modulating intragranule aggregation; however insulin is in a semicrystalline state in the granule [71–75].

## 7. IAPP Has Multiple Physiological Roles

In rats, the circulating concentration of IAPP is reported to be on the order of 3 to 5 picomolar and to rise to 15 to 20 picomolar with elevation of blood glucose [4, 5]. However, the local concentration after release from the granule will be much higher and this is the more relevant number for amyloid formation. The effects of IAPP are receptor mediated, but are still not fully understood. IAPP receptors are generated from coexpression of the calcitonin (CT) receptor with receptor activity-modifying proteins (RAMPs) [76–79]. Interaction with RAMPs changes the specificity of the CT receptor towards IAPP. The CT receptor has two splice variants and there are 3 relevant RAMPs, so there are six different subtypes of IAPP receptors. It is not known whether different receptor subtypes are active in the peripheral tissue and in the CNS. There are no approved small molecule agonists of IAPP receptors.

hIAPP plays a role in maintaining glucose homeostasis, in controlling gastric emptying, and in the suppression of glucagon release [4–6]. The hormone is also involved in controlling satiety and acts as an adiposity signal [80–82]. hIAPP's anorectic effect appears to be mediated mainly at the area postrema of the central nervous system [81]. IAPP has been proposed to help regulate blood glucose levels by inhibiting insulin secretion [83, 84]. Studies conducted with concentrations of hIAPP that are higher than the physiological level have led to suggestions that the polypeptide may inhibit the synthesis of glycogen and insulin-stimulated glucose uptake in isolated rat skeletal muscle [85]. Weight-lowering effects induced by IAPP have been reported in obese rats and humans. Animal studies with food-matched controls have led to the hypothesis that weight loss occurs via mechanisms that are similar to those found with enhanced leptin sensitivity [82, 86, 87]. Several recent reviews provide a more in-depth view of the physiological role of hIAPP [4, 5, 88].

## 8. Monomeric IAPP Does Not Fold to a Compact Structure in Solution, but It Is Not a Random Coil and Can Form Helical Structure on Model Membranes

Monomeric hIAPP does not fold to a compact globular structure and can be classified as an intrinsically disordered protein, but it is not a random coil. NMR chemical shift studies have shown that the region encompassing residues 5–20 of rIAPP and hIAPP transiently sample helical *ϕ*, Ψ angles in solution, but the level of persistent helical structure is low [89, 90]. More persistent helical structure is formed when hIAPP interacts with negatively charged model membranes [90–94]. NMR based structures of IAPP fragments and of full length IAPP in membrane mimetic environments have been reported [92–94]. hIAPP adopts a helix-kink-helix structure on model membranes with the helices located between residues 5 to 17 and 20 to 27. Analysis of peptide fragments has shown interesting differences in the structure of rIAPP and hIAPP in the presence of micelles which are ascribed to the His-18 to Arg substitution in rIAPP. The 1–19 fragments of both peptides adopt similar *α*-helical structures in the presence of detergent micelles, but they bind to micelle in different orientations [93, 95]. hIAPP_1–19_ inserts more deeply into the nonpolar core of the membrane, while rat IAPP_1–19_, with its Arg substitution binds near the surface. hIAPP_1–19_ binds near the surface, similar to rat IAPP_1–19_, at acidic pH when His-18 is protonated, indicating that the net charge of residue 18 is important in controlling the orientation [93, 95]. Membrane-bound structures of full length human and rat IAPP also reveal similarities in the N-terminal half of the molecule, but there are differences in the C-terminal half. The N-terminal portion of both polypeptides adopt *α*-helical structure [92–95]. hIAPP has a partially helical C-terminal region, but the C-terminal region of rat IAPP, with its multiple Pro residues, is disordered [92]. The role of IAPP membrane interactions in amyloid formation and in toxicity is discussed in subsequent sections of this review and more detailed information about membrane bound conformations of IAPP can be found in other reviews in this issue.

## 9. The Parallel, In-Register, *β*-Sheet Architecture of the hIAPP Fibril Has Important Energetic Consequences

Amyloid fibrils form a so-called “cross-*β*” architecture with the interstrand hydrogen bonds oriented parallel to the long axis of the fibril and the *β*-strands oriented perpendicular to the long axis. The parallel, in-register, *β*-sheet structure of amyloids generates effectively infinite arrays of stacked identical residues and this has important energetic consequences. The in-register arrangement implies that there can be significant electrostatic interactions in amyloids. In hIAPP, Arg-11 and His-18 are in the structured core of the fibril, or immediately adjacent to it, arguing that they will make net unfavorable electrostatic contributions to the stability of the fibril. Calculations performed at the level of the linearized Poisson-Boltzmann (PB) approach show that Arg-11 makes significant unfavorable electrostatic interactions but that His-18 does not do so when its side chain is neutral. In this case, the desolvation penalty paid by His-18 is overcome by specific interactions with the imidazole ring [96]. His-18 in hIAPP is replaced by Arg in rIAPP and, based upon the PB analysis, this substitution is expected to destabilize the cross-*β* structure. Consistent with this hypothesis, experimental studies argue that the His-18 to Arg substitution contributes to the inability of rIAPP to form amyloid [31]. A number of groups have independently examined the role of His-18 via pH dependent studies or by computational approaches, and all have concluded that amyloid formation by hIAPP is significantly slowed when the residue is protonated [60, 97–99]. One complication with the simple interpretation of pH dependent studies is that the rate of hIAPP amyloid formation is also affected by the protonation state of the N-terminus [60, 98].

Linearized PB calculations may not be rigorously valid for a strongly coupled system and thus the quantitative details of the analysis should be interpreted with caution. The problem of electrostatic interactions in amyloids has not been studied in detail and seems ripe for further investigation. The pattern of like charges in amyloid fibrils is reminiscent of other systems with repetitive arrangements of charges such as DNA and approaches that have been used to analyze the energetics of DNA should be applicable to amyloids. Unfavorable electrostatic interactions are also likely to arise from the N-terminus of IAPP. Lys-1 is the region of highest charge density in the polypeptide and is expected to make unfavorable electrostatic interactions in the amyloid fibril, even though that region is not part of the well-ordered *β*-sheet core.

The importance of electrostatic interactions in hIAPP amyloid is also reflected in the dependence of the kinetics of hIAPP amyloid formation on ionic strength and on the type of salt. In particular, the rate amyloid formation is significantly accelerated with increasing salt and the effects depend on the choice of anion. An excellent correlation with the anion selectivity series was observed at low and moderate salt concentrations, strongly arguing that anion binding plays a role in the effects [96]. A corollary of this study is that the choice of buffer is expected to impact the rate of hIAPP amyloid formation even when the pH and ionic strength are kept constant. The net positive charge on hIAPP has been exploited to develop charge based inhibitors of amyloid formation and is important for interactions with HSPGs and with anionic membranes (discussed in subsequent sections) [100].

The parallel in-register structure also leads to networks of interactions involving polar uncharged residues. In the fragment model, Ser-28 and Ser-29 are involved in a steric zipper and make extensive hydrogen bonding interactions [26]. Ser-29 in particular forms an interesting network of interpolypeptide hydrogen bonds involving other chains in the same column of monomers as well as interactions with Ser-29 in the symmetry related column (Figure 3). As previously noted, hIAPP contains a large number of Asn residues and the kinetics of amyloid formation are sensitive to isosteric Asn to Leu substitutions. Asn side chains contain both a hydrogen bond donor and acceptor and are hence capable of forming networks of interpolypeptide hydrogen bonds. Asn ladders, hydrogen bonded networks of Asn residues, have been postulated to play an important role in stabilizing amyloid fibrils and MD simulations on a model 5-mer, Asp-Phe-Asn-Lys-Phe, derived from human calcitonin support a role for Asn-Asn stacking interactions in amyloid fibril stability [101].

## 10. *In Vivo* Islet Amyloid Deposits Contain Heparan Sulfate Proteoglycans and Other Factors as well as Incompletely Processed ProIAPP

Islet amyloid contains the heparan sulfate proteoglycan (HSPG) perlecan, apolipoprotein E (apoE), and serum amyloid P component (SAP) [102, 103] in addition to IAPP. There is no correlation between the presence of SAP and islet amyloid deposition. ApoE mouse knockouts do not affect islet amyloid formation and there is no correlation between levels of apoE and islet amyloid formation in hIAPP transgenic mice [103]. This contrasts with the correlation between apoE and amyloid formation in Alzheimer's disease. Secretion of an incompletely processed proIAPP intermediate that includes the N-terminal prosequence, denoted here by Npro-hIAPP, has been reported to be increased in T2D and to be incorporated into islet amyloid [4, 62, 63].

## 11. Model Glycosaminoglycans and Model Membranes Containing Anionic Lipids Accelerate IAPP Amyloid Formation* In Vitro*

hIAPP is cationic and ionic interactions facilitate its binding to negatively charged membranes, negatively charged biopolymers, and negatively charged surfaces. It is not known if perlecan is associated with islet amyloid because* in vivo* amyloid fibers are long-lived structures that present HSPG binding sites, or because HSPGs directly promote amyloid formation, but it is well documented that the glycosaminoglycan (GAG) chains of HSPGs catalyze hIAPP amyloid formation* in vitro* [64, 104]. There is indirect evidence that HSPGs promote islet amyloid formation* in vivo*. Overexpression of heparanase in a double transgenic mouse model that overexpresses hIAPP reduced the amyloid load, while inhibition of GAG synthesis reduced hIAPP amyloid deposition in cultured islets [105, 106].

The factors that trigger islet amyloid formation* in vivo* are still mysterious, but one model postulates binding of the Npro-hIAPP processing intermediate to the GAG chains of perlecan [61, 62, 64]. The N-terminal extension actually makes hIAPP less amyloidogenic and more soluble but enhances interactions with GAGs. In this model, incompletely processed hIAPP binds to HSPGs, thereby leading to a high local concentration of the peptide which promotes aggregation and amyloid fibril formation. The resulting aggregates then recruit more Npro-hIAPP and fully processed IAPP. In support of the model, interactions with model GAGs have been shown to accelerate NproIAPP amyloid formation* in vitro* and the resulting fibrils can seed amyloid formation by fully processed mature hIAPP [64].

Anionic model membranes promote hIAPP amyloid formation* in vitro* and more highly charged systems have a larger effect for experiments conducted at high peptide to lipid ratios [107]. The mechanism of membrane catalyzed hIAPP aggregation is not completely understood, but helical intermediates are thought to be important [90, 91, 107–109]. Much of the work on hIAPP-membrane interactions has used model membranes comprised of pure anionic lipids, such as phosphatidylglycerol or phosphatidylserine, or mixtures of anionic lipids with zwitterionic lipids, such as phosphocholine. The content of anionic lipid typically ranges from 50 or more to 20 mole % in these systems, which is much higher than that found in *β*-cells [110]. The phospholipid composition of the *β*-cell is also very different from most model systems. In addition, *β*-cell membranes contain cholesterol and the *β*-cell plasma membrane is asymmetric with the anionic lipids localized on the inner leaflet. These considerations naturally lead to the question of how well model membranes recapitulate the situation* in vivo*. Model membranes made up of the phospholipids found in *β*-cell membranes, but lacking cholesterol, also accelerate hIAPP amyloid formation, as do anionic model membranes that are capable of forming lipid rafts [110–112]. The effects of cholesterol have also been examined [113].

## 12. Does Islet Amyloid Have an Extracellular or Intracellular Origin?

The initiation site for islet amyloid formation* in vivo* is controversial and there are conflicting reports in the literature. Amyloid deposits detected in T2D appear to be extracellular and initial studies with transgenic mice were consistent with extracellular deposition. However other studies with rodent models in which IAPP is overexpressed are consistent with an intracellular origin [4, 114]. It is worth bearing in mind that some transgenic mouse models have high copy numbers of the human IAPP gene and can produce high levels of hIAPP. Significant overproduction of the polypeptide could play a role. In contrast to the mouse studies, a recent investigation used a cultured transgenic islet model to show that secretion of IAPP is an important factor in *β*-cell toxicity and islet amyloid formation. Two complimentary sets of reagents were employed: one that inhibited IAPP secretion, but maintained the level of production of IAPP and a second that increased IAPP secretion but did not increase the amount of IAPP produced. Inhibiting IAPP secretion reduced amyloid formation, but increasing secretion increased toxicity and amyloid formation [115]. The results are consistent with an extracellular origin of islet amyloid. The differences between the various studies might be related to the level of production of hIAPP [4, 114–116]. Clarifying whether islet amyloid has an intracellular or extracellular origin is important since the answer might impact therapeutic strategies and drug design.

## 13. IAPP Toxicity Impacts Type-2 Diabetes and Islet Cell Transplantation

Interest in islet amyloid has undergone resurgence due to the realization that *β*-cell dysfunction and death and the loss of *β*-cell mass are key features of T2D [117, 118]. *β*-cell dysfunction and the decline in *β*-cell mass are attributed to several factors, including glucolipotoxicity, inflammation, accumulation of cholesterol, and islet amyloid formation [117–122].

Islet amyloid deposition is also a key factor contributing to the failure of islet cell transplants. Islet amyloid has been detected in transplanted human islets in a patient that suffered islet graft failure and has been shown to form rapidly after transplantation of human islets into nude mice [4, 123–125]. Islet amyloid formation in the mouse studies is correlated with the loss of *β*-cells and occurs before the recurrence of hyperglycemia. Porcine IAPP is far less amyloidogenic than hIAPP and the prevention of amyloid formation by transplantation of porcine islets prolongs islet graft survival [126]. Recent work also highlights a role for hIAPP aggregation and hyperamylinemia in cardiovascular complications of diabetes [16, 127].

## 14. Multiple Mechanisms of hIAPP Induced *β*-Cell Toxicity Have Been Proposed

A range of mechanisms have been proposed to account for the toxic effects of amyloidosis, but the exact causes of cell death are still not completely defined. In some cases, amyloid fibril deposits disrupt tissue and can lead to organ failure, but, in most cases, activation of overlapping cellular mechanisms and downstream signaling pathways are believed to lead to toxicity. These include receptor-mediated mechanisms and non-receptor-mediated processes.

ER stress, defects in autophagy, the enhanced production of proinflammatory cytokines, mitochondrial membrane damage, permeabilization of cell membranes, activation of Calpain-2, receptor-mediated mechanisms linked to oxidative stress, and the activation of cell death signaling pathways have all been proposed to contribute to IAPP toxicity [128–147]. Here we provide an overview; more information can be found in other recent review articles [4, 15, 116, 130].

Defects in autophagy play a role in the toxicity of other amyloidogenic proteins. Upregulation of autophagy is a common protective response to the accumulation of toxic amyloidogenic aggregates in degenerative disease. However, autophagocytosis and lysosomal degradation of amyloidogenic polypeptides are not always entirely successful and the resulting accumulation of amyloidogenic aggregates can lead to autophagy-mediated lysosomal dysfunction and cell death. Overexpression of hIAPP in *β*-cells has been reported to lead to impaired autophagy [135, 143, 146]. Inhibition of autophagy-lysosomal degradation has been shown to enhance hIAPP induced *β*-cell apoptosis, while stimulation of autophagy protected against IAPP toxicity [135, 146].

Defects in ER stress, endoplasmic reticulum associated protein degradation (ERAD), and the unfolded protein response (UPR) have all been reported to induce *β*-cell death by hIAPP aggregates. ProIAPP and partially processed proIAPP may be one of deleterious species in cases where toxicity arises from intracellular aggregates since proIAPP miss-processing has been shown to occur in diabetes and posttranslational modification is completed in the Golgi and insulin secretory granules [4, 7, 8]. The role of ER stress in hIAPP mediated toxicity* in vivo* is controversial. Transgenic mouse studies using mice that overexpress hIAPP led to the proposal that ER stress is a key contributor to hIAPP induced *β*-cell dysfunction and exogenously added hIAPP has been reported to induce ER stress [114, 141]. On the other hand, ER stress was not detected in studies of cultured islets that produce IAPP at more physiological levels [142].

Chronic inflammation may be an important contributing factor to amyloidosis toxicity and it is frequently observed in local and systemic amyloidosis. hIAPP aggregates can contribute to *β*-cell dysfunction by triggering a localized inflammatory response by stimulating inflammasome activity [136, 137]. Inflammasomes are multiprotein assemblies that recognize a diverse range of proinflammatory stimuli and produce active caspase-1. Caspase-1 in turn activates the cytokines IL-1*β* and IL-18 by cleaving their proforms. IL-1*β* is thought to play a direct role in hIAPP induced *β*-cell death and dysfunction [136, 137].

Amyloid formation by hIAPP induces apoptosis in cell culture and in isolated human islets, but the pathways that lead to IAPP induced *β*-cell apoptosis are not yet fully elucidated [128–134]. The JNK pathway mediates *β*-cell apoptosis in islets and in cultured cells exposed to high concentrations of hIAPP and is upregulated in response to amyloid formation by endogenous hIAPP [133]. Interaction of exogenous or endogenous hIAPP aggregates with Fas, also known as the death receptor, leads to caspase-3 activation, while deletion of Fas protects *β*-cells from hIAPP induced toxicity and inhibition of caspase-3* in vivo* protects *β*-cells from hIAPP induced *β*-cell apoptosis [15, 134].

IAPP toxicity has also been proposed to result from the perturbation of membrane integrity [144, 145, 148]. The efficiency with which hIAPP permeabilizes membranes depends on a range of factors including the lipid to peptide ratio, the lipid composition, pH, and ionic strength. IAPP interacts significantly more strongly with model membranes that contain a high fraction of anionic lipids. Commonly used model systems contain a much higher percentage of anionic lipids than that found in the *β*-cell membrane [110] and usually lack cholesterol and gangliosides. These are important considerations since high percentages of anionic lipids significantly promote IAPP membrane interactions and because gangliosides and cholesterol modulate hIAPP membrane interactions [111, 148]. In addition, there is experimental evidence that membrane gangliosides and cholesterol play a role in the uptake and clearance of hIAPP [111, 148]. More physiologically relevant model membrane systems are starting to be employed in biophysical investigations and should provide new insights [110–112].

The correlation between* in vitro* biophysical studies using model membranes and* in vivo* toxicity is not clear and caution needs to be employed when extrapolating from biophysical studies that utilize simple model membranes to the* in vivo* environment. For example, variants of hIAPP that do not induce *β*-cell death* in vivo* and are not toxic* in vitro* can disrupt standard model membranes* in vitro* and can do so as effectively as hIAPP [149]. It is also worth noting that exogenously added IAPP has been reported to induce different toxic effects on closely related cell types, arguing that nonspecific membrane disruption cannot be the only mechanism of toxicity [150].

Mechanistic studies of IAPP induced model membrane disruption are an active area of research and a variety of models have been proposed. Some studies provide evidence for a detergent or carpeting mechanism while others have argued for a pore-like mechanism. The process of fiber growth at the membrane surface can contribute to membrane disruption in some cases, while other studies have shown that formation of *β*-structure is not required to disrupt membranes [149, 151–156]. It is possible that multiple mechanisms are operative and the specific mechanism depends on the membrane system under investigation [157, 158]. Much more information on the mechanisms of membrane disruption can be found in other contributions to this issue.

## 15. Macromolecular Crowding Impacts the Rate of Amyloid Formation and Does So by Multiple Mechanisms

The cell is an inherently crowded environment, containing numerous proteins and other macromolecules as well as osmolytes, and there is no guarantee that a protein will behave the same in dilute solution and in a crowded environment. The effects of molecular crowding and osmolytes on the stability and folding of globular proteins are well studied and are still an active area of research. Early work focused on inert crowders and the role of excluded volume effects, but more recent efforts have been directed at better mimics of the cellular environment and consideration of interactions beyond excluded volume [159–163]. Indeed, it is now clear that the effects of many crowding agents, and by implication the cellular environment, cannot be explained solely on the basis of excluded volume. Less work has been reported on the effects of crowding and osmolytes on amyloid formation, but this is an active area and recent papers have appeared that have examined the effects of osmolytes and crowders on amyloid formation by hIAPP and other proteins [164–169]. Two themes which have emerged from recent studies are that the impact of crowding on amyloid formation goes beyond excluded volume effects and specific interactions of the amyloidogenic protein of interest with biologically relevant crowding agents can make significant contributions. The effects of crowding agents and osmolytes on amyloid formation by IAPP are reviewed in detail in this volume by Gao and Winter [169].

## 16. Nontoxic Bioactive Variants of IAPP with Improved Solubility Are Clinically Relevant for the Treatment of Type-1 Diabetes and Obesity

**Figure 4:**
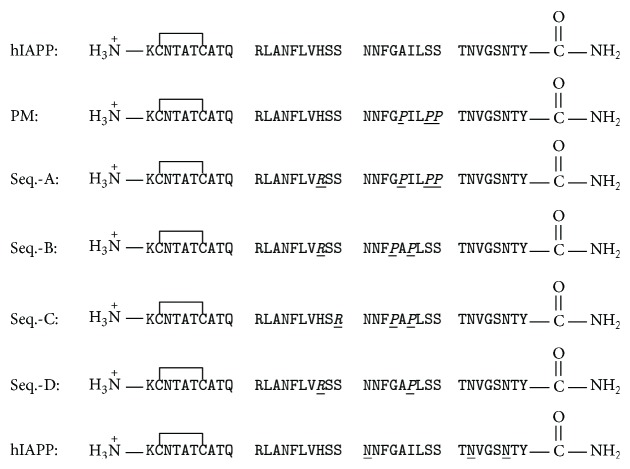
Primary sequences of human IAPP, Pramlintide, and four rationally designed sequences with improved solubility, denoted by Seq-A through Seq-D. The final sequence, denoted by hIAPP ^*∗*^, represents a family of designed bioactive analogs of human IAPP in which one of the Asn residues indicated with a ( ^*∗*^) has been glycosylated.

Coadministration of IAPP with insulin helps to normalize fluctuating glucose levels to a greater degree than is possible with insulin alone; however the extreme tendency of IAPP to aggregate and its amyloidogencity and toxicity prevent its direct use as an adjunct to insulin therapy [170–174]. A nonamyloidogenic analog of human IAPP, denoted by Pramlintide, in which residues 25, 28, and 29 were replaced by proline is approved by the FDA for use in type-1 and type-2 diabetes [170, 171]. Pramlintide was designed based on comparison of the sequences of rat and human IAPP and is simply human IAPP with the three Pro substitutions found in the rat polypeptide. Ideally, Pramlintide would be formulated with insulin and coadministrated. Unfortunately, Pramlintide is not soluble at the appropriate pH. There is also interest in combining leptin and hIAPP for the treatment of obesity [175]. However, coformulation is also difficult in this case because of the poor solubility of hIAPP and Pramlintide. Recent efforts at developing more soluble analogs of hIAPP have utilized a number of approaches. Nontoxic variants of hIAPP with considerably improved solubility at pH 7.4 have been developed by rationally redesigning the sequence to incorporate strategic proline residues together with additional charges (Figure 4) [176]. An alternative approach has employed the strategy of conjugating groups to hIAPP. Engineering the polypeptide by the selective modification of specific Asn residues with carbohydrates or by attaching polyethylene glycol to the side chain and N-terminal amino group of Lys-1 has led to bioactive analogs with improved properties [177, 178]. An interesting feature of the Asn modification work is that the effects of the modifications on the normal activity of hIAPP were found to depend on the site modified and thus provide indirect information about regions of hIAPP which are critical for receptor binding [177]. A fourth approach has made use of N-methylation and builds upon the development of N-methylated hIAPP analogs as potent inhibitors of wild type aggregation and toxicity [34]. N-methylated analogs have been reported which are bioactive and nonamyloidogenic and which inhibit amyloid formation by insulin [179, 180]. Collectively, these different approaches demonstrate that there is considerable potential for the design of hIAPP therapeutics with improved properties.

## 17. Conclusions

Impressive progress has been made in studies of amyloid formation by hIAPP, but important challenges remain. These include identifying the initiation site(s) of amyloid formation* in vivo*; defining the nature of the toxic species; elucidating the mechanisms of islet amyloid formation* in vivo* and* in vitro*; understanding the mechanisms of *β*-cell death; defining the mechanisms of hIAPP clearance* in vivo* and the role such processes may play in IAPP toxicity. Experimental challenges include relating reductionist biophysical experiments to the situation* in vivo* and understanding the connection between mouse models that highly overexpress hIAPP and human *β*-cell physiology. Although not discussed in this review, the development of inhibitors of hIAPP toxicity is also an area that warrants further effort. There are no clinically approved inhibitors of IAPP toxicity and very few, if any, effective “drug-like” inhibitors of IAPP amyloid formation have been reported in the literature.

## Abbreviations

Ala: Alanine
apoE: Apolipoprotein E
Asn: Asparagine
Arg: Arginine
CGRP: Calcitonin gene-related peptide
GAG: Glycosaminoglycan
His: Histidine
HSPG: Heparan sulfate proteoglycan
IAPP: Islet amyloid polypeptide
hIAPP: Human IAPP
rIAPP: Rat IAPP
Leu: Leucine
Lys: Lysine
Phe: Phenylalanine
PB: Poisson-Boltzmann
Pro: Proline
SAP: Serum amyloid P component
Ser: Serine
TFA: Trifluoroacetic acid
Thr: Threonine
Tyr: Tyrosine
Val: Valine
2DIR: Two-dimensional infrared.
